# Lack of association between mutation in IL36RN and palmoplantar pustular psoriasis in Chinese patients^☆^^☆☆^

**DOI:** 10.1016/j.abd.2019.01.008

**Published:** 2019-10-26

**Authors:** Yu Xiaoling, Shu Dan, Jin Hongzhong

**Affiliations:** Department of Dermatology, Peking Union Medical College Hospital, Chinese Academy of Medical Sciences, Peking Union Medical College, Beijing, China

**Keywords:** Genes, Mutation, Psoriasis

## Abstract

**Background:**

Palmoplantar pustulosis is considered to be a localized pustular psoriasis confined to the palms and soles. Mutation of the IL36RN gene, encoding interleukin-36 receptor antagonist (IL-36Ra), is associated with generalized pustular psoriasis, but IL36RN mutations in Chinese palmoplantar pustulosis patients have not previously been investigated.

**Objective:**

The aim of this study was to evaluate the mutation of IL36RN in Chinese patients with palmoplantar pustulosis.

**Methods:**

Fifty-one Han Chinese patients with palmoplantar pustulosis were recruited. All exons and exon-intron boundary sequences of IL36RN were amplified in polymerase chain reactions, and Sanger sequencing of the amplicons was performed.

**Results:**

Among the 51 palmoplantar pustulosis patients, four different single-base substitutions were identified in nine patients. The mutations were c.140A>G/p.Asn47Ser in five patients, c.258G>A/p.Met86IIe in two patients, and c.115+6T>C and c.169G>A/p.Val57IIe in one patient each. All mutations were heterozygous. Comparison with the human genome database and reported literature suggested that these variants may not be pathogenic mutations causing palmoplantar pustulosis. Furthermore, there was no difference in disease severity, onset age, or disease duration between patients with these heterozygous IL36RN variants and those without (*p* > 0.1).

**Study limitation:**

Lack of the further evaluation of IL36Ra protein in palmoplantar pustulosis lesions.

**Conclusions:**

The four variants of IL36RN identified did not appear to be associated with the specific phenotypes of palmoplantar pustulosis.

## Introduction

Palmoplantar pustulosis (PPP) is a chronic, relapsing, inflammatory skin disease that is restricted to the palms and soles. PPP appears more frequently in middle-aged adults and women, and usually develops resistance to therapy.^1^ Various factors such as metal allergy, cigarette smoking, infection, and thyroid disease are associated with PPP.^2^, ^3^ Erythema, sterile pustules, and scales are common clinical features. Histopathology shows parakeratosis, psoriasiform epidermal hyperplasia, spongiosis, and pustules filled with neutrophils. PPP shares many clinical and histological features with generalized pustular psoriasis (GPP), thus PPP is considered to be a localized type of GPP.^4^ GPP presents as edematous erythema, with sterile pustules all over the body.

It is known that GPP in patients with a familial history and without psoriasis vulgaris is mainly caused by homozygous or compound heterozygous mutations of the IL36RN gene, which encodes an antagonist of IL-36 isoforms, including IL-36α, IL-36β, and IL-36γ.^5^ IL-36 triggers a proinflammatory reaction *via* the mitogen-activated protein kinase and nuclear factor-κB signaling pathways. Mutation of IL36RN results in structural and functional deficiency of IL-36Ra, which then cannot inhibit the abnormal inflammatory reaction mediated by IL-36 and its receptor.^6^ The relationship between IL36RN and PPP has also been studied, but the results are controversial. Research in Japan and Europe has suggested that PPP has no association with IL36RN mutation.^7^, ^8^ However, another European study indicated that GPP and PPP underlie a spectrum of IL36RN variant-associated pustular psoriasis.^9^ The present study aimed to investigate the association between IL36RN and PPP in Chinese patients, to determine the relationship between IL36RN and PPP and to contribute data elucidating the role that IL36RN plays in pustular disease in different ethnicities.

## Methods

### Patients

Fifty-one Han Chinese patients with PPP in the active disease stage who visited the Department of Dermatology at Peking Union Medical College Hospital between 2014 and 2016 were included in this study, diagnosed by the same two dermatological specialists according to their clinical manifestation, with or without histopathology. Pregnant and SAPHO (synovitis, acne, pustulosis, hyperostosis, and osteitis) syndrome patients were excluded. Disease severity for every patient was assessed using the Palmoplantar Pustular Psoriasis Area and Severity Index (PPPASI), which evaluates the area of lesion involvement (score: 0 = none, 0 < 1 ≤ 10%, 10% < 2 ≤ 30%, 30% < 3 ≤ 50%, 50% < 4 ≤ 70%, 70% < 5 ≤ 90%, 90% < 6 ≤ 100%) and the severity for each of the three clinical skin signs: erythema (E), pustules (P), and desquamation (D) (score: 0 = none, 1 = slight, 2 = moderate, 3 = severe, 4 = very severe).^10^ The final PPPASI was calculated by (E + P + D) area × 0.2 (right palm) + (E + P + D) area × 0.2 (left palm) + (E + P + D) area × 0.3 (right sole) + (E + P + D) area × 0.2 (left sole), which ranges from 0 to 72. Patients’ demographic characteristics, PPP onset age, disease duration, and family history were determined in interviews. All patients gave written informed consent. The study was approved by the Medical Ethics Committee of Peking Union Medical College Hospital; this work was conducted according to the Declaration of Helsinki principles.

### Mutation detection

Blood samples were collected from all 51 patients. Genomic DNA was extracted from peripheral blood leukocytes using the Axy Prep Blood Genomic DNA Miniprep Kit (Axygen Biosciences, Tewksbury, MA, United States). The IL36RN gene sequence was obtained from GenBank (NM_012275). Seven pairs of primers for the polymerase chain reaction (PCR) amplification of IL36RN for were designed using Primer 5 (http://primer5.ut.ee/) and synthesized by the Majorbio Company (Shanghai, China; Table 1). All five IL36RN coding exons, as well as their flanking introns, were amplified by PCR using the extracted genomic DNA as template. The total volume of the reactions was 50 μL: 25 μL of 2× EasyTaq PCR Super Mix, 4 μL of DNA, 2 μL of forward primer, 2 μL of reverse primer, and 17 μL of double-distilled water. PCR conditions were as follows: initial denaturation at 94 °C for 5 min; 35 cycles of denaturation at 94 °C for 30 s, annealing at 60 °C for 30 s, and elongation at 72 °C for 1 min; final elongation at 72 °C for 7 min. The amplified PCR products were verified by agarose gel electrophoresis and observation under ultraviolet light. DNA purification and sequencing were performed by the Majorbio Company. The likely function of mutant protein sequences was assessed using the SIFT and/or PolyPhen pathogenicity prediction tools.

**Table 1:** IL36RN exon primer sequences and polymerase chain reaction (PCR) amplicon lengths.

	Forward primer (5′–3′)	Reverse primer (5′–3′)	bp
IL-36RN-1	AGTGCTTCTGGCGACTTAGG	GGAGAGAGAGGCTGAGTTGG	276
IL-36RN-2	CTGACCCCAGACCCAGAATC	AGCTGGACAACGGGTCTATC	261
IL-36RN-3	GTTACTTCTGGCACAGTAGG	CACTTTGCTGAGAGGTGTAG	392
IL-36RN-4	TCATGACAGCTGCTGAGAAG	AGCTGCCATCAACAGAATCC	386
IL-36RN-5.1	AGATGCTGAGCCTACTGAAG	TCTGACATCAGCACCTCCTC	950
IL-36RN-5.2	TAGAGTCAGGGATCTATGGC	GTGTCCTCTCCTTTTCATAC	940
IL-36RN-5.3	CAAATTCACATCCTTCTTGG	GGGTAAATGAAGGATAGTTG	917

bp, base pair.

### Statistical analysis

Statistical software (SPSS v. 20.0; SPSS Inc – Chicago, IL, United States) was used to analyze the data in this study. Means and standard deviations were compared between patients with and without an IL36RN mutation, using Student's *t*-test for independent samples. When the data did not follow a normal distribution, the Mann–Whitney *U*-test was used to calculate statistical significance. A two-class logistic regression model was applied to compare patients with and without heterozygous IL36RN mutations, adjusted by risk factors (sex, age, family history of PPP). The significance level was set at *p* < 0.05.

## Results

There were 35 women and 16 men recruited in this study. The patients’ mean age was 48.9 ± 11.9 years, and the mean age of PPP onset was 45.0 ± 11.9 years. The mean duration of PPP was 38.7 ± 62.2 months. No patients had a family history of PPP. The mean PPPASI score of all patients was 15.1 ± 10.9 (Table 2).

**Table 2:** Demographic characteristics of palmoplantar pustulosis (PPP) patients.

	Number of patients	Family history	Mean age	Mean onset age of PPP	Median (25th percentile, 75th percentile) disease duration (months)	Mean PPPASI
Total (male/female)	51 (16/35)	0	48.9 ± 11.9 (range: 26–78)	45.0 ± 11.9 (range: 24–63)	14 (6, 36)	15.1 ± 10.9 (range: 1.2–49.0)
Patients with heterozygous IL36RN mutations	9 (2/7)	0	52.5 ± 8.4 (range: 40–66)	48.7 ± 7.1 (range: 40–62)	12.5 (6, 45)	10.4 ± 9.2 (range: 1.2–28.9)
Patients without heterozygous IL36RN mutations	42 (14/28)	0	48.1 ± 12.3 (range: 26–78)	44.3 ± 12.6 (range: 24–63)	26 (4, 35)	16.1 ± 11.1 (range: 2.1–49.0)

PPPASI, Palmoplantar Pustular Psoriasis Area and Severity Index.

**Figure 1:**
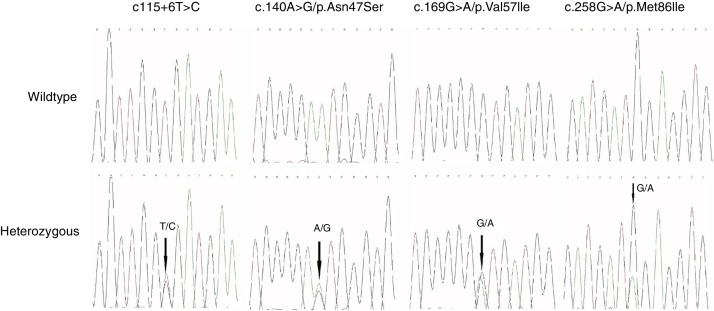
Sequence chromatogram of the heterozygous variants of IL36RN in palmoplantar pustulosis.

Four distinct IL36RN mutations were identified among nine of the 51 PPP patients. The four non-synonymous, single nucleotide variants included c.140A>G/p.Asn47Ser in five patients, c.169G>A/p.Val57IIe in one patient, and c.258G>A/p.Met86IIe in two patients. The fourth mutation was c.115+6T>C in intron 3, found in one patient. All mutations were heterozygous, and no patient had a homozygous or compound heterozygous mutation. The electropherograms of all variants are shown in Fig. 1.

As a result of the functional predictions, p.Asn47Ser and p.Met86IIe were classified as probably damaging by PolyPhen, but as tolerated by SIFT. p.Val57IIe was classified as benign and tolerated by both PolyPhen and SIFT.

**Figure 2:**
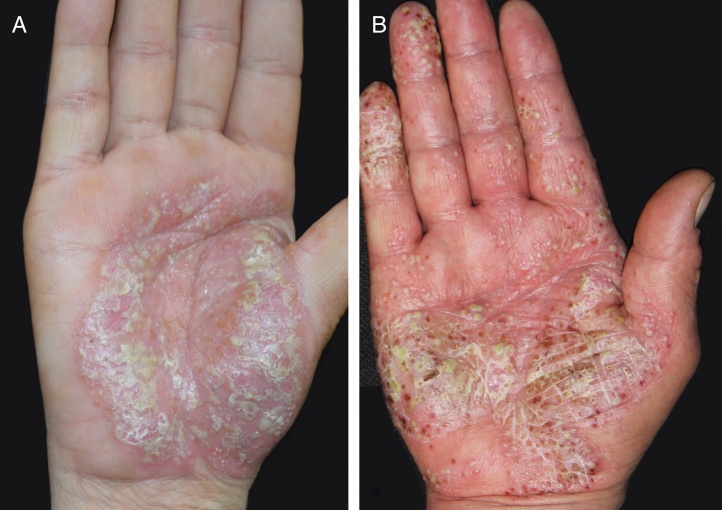
Clinical picture: palmoplantar pustulosis rash on hands: (A) patient with c.140A>G/p.Asn47Ser heterozygous mutation; (B) patient with no mutation of IL36RN.

The mean ages of PPP onset with and without IL36RN heterozygosity were 48.7 ± 7.1 and 44.3 ± 12.6 years, respectively. There was no significant difference between these two groups (*p* = 0.378). The median (25th percentile, 75th percentile) disease duration was 14 (6, 36) months for all PPP patients, 12.5 (6, 45) months for patients with heterozygous IL36RN variants, and 26 (4, 35) months for patients without. Again, no statistically significant difference was observed (*W* = 165.5, *p* = 0.711). The mean PPPASI scores were 10.4 ± 9.2 for patients with heterozygous IL36RN mutations and 16.1 ± 11.1 for patients without, with no significant difference between them (*p* = 0.219). There were still no differences among PPPASI score, disease duration, and onset age between patients with or without IL36RN mutation after adjusting for sex, age, and family history of PPP (all *p* > 0.1). The clinical manifestations of two patients with or without IL36 RN mutations are shown in Fig. 2.

## Discussion

The IL36RN gene is located on the long arm of chromosome 2 and consists of five exons, encoding the IL-36 antagonist, IL-36Ra.^11^, ^12^ IL-36Ra inhibits the IL-36-mediated inflammatory response by binding to the IL-36 receptor.^13^ Pathogenic mutations of IL-36RN cause diseases such as GPP.^6^, ^14^, ^15^ PPP is regarded as a localized form of GPP.^4^

It has been shown that many cases of GPP are caused by mutations of the IL36RN gene.^16^ However, there have been only three studies of the IL36RN mutation in PPP to date, conducted on various ethnic groups. Setta-Kaffetzi et al. found that seven PPP patients harbored a p.Ser113Leu variant and four were homozygous, which are similar mutation rates to those found for GPP in their study, indicating that the IL36RN alleles were associated with a phenotypic profile that including PPP and GPP.^9^ In contrast, Mössner et al. found four patients with heterozygous IL36RN variants who were without pathogenicity among 251 PPP patients, which suggested that PPP was not associated with loss-of-function IL36RN mutations in Europeans.^8^ Takahashi et al. found four of 88 Japanese PPP patients had heterozygous IL36RN variants, and similarly concluded that these variants did not seem to be associated with the phenotype of PPP.^7^ The different results in these reports may reflect the different ethnicities of the study populations. Before now, there have been no studies of IL36RN mutation in Chinese PPP patients. The present study indicated that there may be no association between mutation in IL36RN and palmoplantar pustulosis in Chinese patients.

Fifteen distinct mutations have been identified in GPP patients, with p.Ser113Leu, p.Leu27Pro, and c.115+6T>C the most common variants in European, African, and Asian populations, respectively.^17^ c.115+6T>C as well as c.227T>C were identified as the founder IL36RN mutations in studies of Chinese patients.^5^, ^17^

In the present study, four IL36RN variants were identified in Chinese PPP patients. A heterozygous c.140A>G/p.Asn47Ser variant was found in five patients (9.8%). According to data derived from the UCSC website of the 1000 Genomes Project, there are 66 among 5008 (1.3%) healthy individuals carrying this variant. Li et al. suggested that among 373 healthy Chinese subjects, 25 (6.7%) carried p.Asn47Ser, of whom 24 were heterozygous and one was homozygous.^17^ Hayashi et al. reported that among 1120 healthy Japanese subjects, 76 had a heterozygous p.Asn47Ser variant and one had a homozygous variant (6.9% in total).^18^ Furthermore, the frequency of p.Asn47Ser carriers among 111 Chinese psoriasis vulgaris patients has been reported as 9.0%; thus, p.Asn47Ser is likely to be a polymorphism in PPP.^17^

The most common mutant genotype in Asian GPP patients from China, Japan, and Malaysia is c.115+6T>C.^16^, ^17^, ^19^ One patient (2.0%) in the current study and seven of 168 (4.2%) healthy controls in a previous study by the present authors harbored this heterozygous variant.^20^ Furthermore, Li et al. found that the frequency of c.115+6T>C was 3.6% in healthy controls and 1.8% in psoriasis vulgaris patients, higher than that in the PPP group of the current study.^17^

The c.169G>A/p.Val57IIe and the c.258G>A/p.Met86IIe were detected in one and two patients respectively, and have not previously been reported. From the available data, heterozygous p.Val57IIe or p.Met86IIe variants may not be associated with the manifestation of GPP or PPP. p.Val57IIe was classified as benign and tolerated by PolyPhen and SIFT, and p.Met86IIe was classified as tolerated by SIFT. Thus, these variants may not be pathogenic mutations underlying PPP.

Overall, there was no difference in severity, mean onset age, or disease duration between patients with heterozygous IL36RN variants and those without. IL36RN may have no association with the clinical manifestation of PPP. Another piece of evidence is that the skin lesions of heterozygous IL36RN PPP patients stained positively for IL-36Ra in an immunohistochemical analysis, showing no difference with normal controls.^7^ Moreover, Onoufriadis et al. concluded that three GPP patients had homozygous (p.Ser113Leu) or compound heterozygous mutations (p.Ser113Leu, p.Arg48Trp), but no mutation was observed in another two GPP patients with PPP complications.^14^ These authors speculated that IL36RN is implicated in GPP but is not associated with PPP, which is consistent with the conclusion of the current study. Hence, the heterozygous variants of IL36RN herein described may not be pathogenic factors in PPP. Another hypothesis indicated that heterozygous or compound heterozygous mutations of IL36RN may be a predisposing factor for GPP with psoriasis vulgaris because three of 20 GPP patients with psoriasis vulgaris complications were found to carry heterozygous IL36RN variants.^16^ However, whether PPP patients with an IL36RN variant have a higher risk developing GPP is unknown, and needs further investigation.

## Conclusion

In conclusion, the four different IL36RN variants detected in this study of 51 Chinese patients are not likely to be associated with the pathogenesis of PPP. These results are in concordance with European and Japanese studies suggesting that IL36RN mutations are not the pathogenic factor underlying PPP.

## Financial support

National Natural Science Foundation of China 81773331; Capitals Funds for Health Improvement and Research 2016-2-4018.

## Author's contribution

Yu Xiaoling: Statistical analysis; approval of the final version of the manuscript; conception and planning of the study; elaboration and writing of the manuscript; obtaining, analyzing and interpreting the data; intellectual participation in propaedeutic and/or therapeutic conduct of the cases studied; critical review of the literature.

Shu Dan: Approval of the final version of the manuscript; conception and planning of the study; effective participation in research orientation; critical review of the manuscript.

Jin Hongzhong: Approval of the final version of the manuscript; conception and planning of the study; effective participation in research orientation; intellectual participation in propaedeutic and/or therapeutic conduct of the cases studied; critical review of the manuscript.

## Conflicts of interest

None declared.
